# Evaluation of the Frequency and Patterns of Cervical Cancer Recurrence After Treatment Using ^18F‐FDG PET‐CT: A Cross‐Sectional Study

**DOI:** 10.1002/hsr2.71566

**Published:** 2025-11-23

**Authors:** Babar Ali

**Affiliations:** ^1^ Department of research Medical Research Circle (MedReC) Bukavu DR Congo

**Keywords:** ^18F‐FDG PET‐CT, adenocarcinoma, cervical cancer, metastasis, recurrence, squamous cell carcinoma

## Abstract

**Background and Aim:**

Cervical cancer recurrence remains a major clinical challenge, particularly in patients with advanced disease or high‐risk pathological features. This study aimed to evaluate the frequency and distribution of recurrence and metastases in posttreatment cervical cancer patients using ^18F‐FDG PET/CT, with additional analysis by histological subtype and primary treatment modality.

**Methods:**

This retrospective cross‐sectional study included 120 posttreatment cervical cancer patients who underwent ^18F‐FDG PET/CT at the Institute of Nuclear Medicine and Oncology, Lahore, Pakistan, between July 2024 and January 2025. Patient demographics, tumor histology, and PET‐CT findings were recorded. Recurrence was classified as local, lymphatic, visceral, or skeletal. Data were analyzed using SPSS (v26.0). The Chi‐square test compared recurrence patterns between histological subtypes. A *p*‐value ≤ 0.05 was considered significant.

**Results:**

The overall recurrence rate was 65.0% (*n* = 78). Local recurrence occurred in 33.3%, and lymph node metastases were most frequent in the chest (40.8%), followed by abdominal (25.0%), pelvic (21.7%), and neck (10.8%) nodes. Visceral metastases involved the lungs (17.5%) and liver (14.2%), while skeletal metastases were uncommon (8.3%). Squamous cell carcinoma (73.3%) was the predominant subtype, but adenocarcinoma carried a significantly higher relative risk of recurrence (*p* = 0.041). Treatment modality influenced outcomes, with the lowest recurrence after surgery alone (44.0%) and higher recurrence after chemoradiation (70.6%) or combined therapy (70.4%).

**Conclusion:**

This study demonstrates that ^18F‐FDG PET‐CT is a valuable imaging modality for detecting recurrence and metastases in posttreatment cervical cancer patients. The recurrence rate was high, with adenocarcinoma showing a greater risk compared to squamous cell carcinoma. PET‐CT effectively identified both local and distant disease, with chest and abdominal nodes, lungs, and liver being the most frequent metastatic sites, while skeletal involvement was rare. Recurrence was also significantly associated with treatment modality, being lowest after surgery and higher among patients treated with chemoradiation or combined therapy.

## 1 Introduction

Cervical cancer is a cancer of the female reproductive system that originates in the cervix. It is the fourth leading cause of cancer in women worldwide after breast, lung, and colorectal cancer and the third most common neoplasm among women in Pakistan [1, 2, 3]. According to a World Health Organization (WHO) report issued in 2024, 350 000 people died globally in 2022 [2]. Annually, around 5008 women in Pakistan are diagnosed with cervical cancer, resulting in a fatality rate of 3197 [3]. Given the high prevalence of cervical cancer, early detection and timely intervention are essential to improve outcomes. It is a largely preventable disease, owing to the universal application of Papanicolaou smear screening, which has resulted in the early diagnosis of precancerous tumors that can be treated before they metastasize [4]. However, due to a lack of extensive screening and vaccination programs, it remains a prevalent genital cancer seen in clinical practice in women from low‐ and middle‐income countries (LMICs), contributing to higher morbidity and mortality rates [5]. Persistent infection with high‐risk human papillomavirus (HPV) is the leading cause of cervical cancer, resulting in malignant transformation of cervical epithelial cells [6, 7]. The other minor risk factors include smoking, inherited factors, a compromised immune system, taking birth control pills, starting sex at a young age, and having many sexual partners [8]. Advances in early detection and medical treatment have significantly enhanced survival rates; yet, recurrence remains a major issue in illness management. Recurrent cervical cancer can manifest as local tumor relapse, lymphatic spread, or distant metastases, and it usually occurs within the first 2 years of initial treatment. Given its aggressive nature and poor prognosis, early detection of recurrence is crucial for better patient outcomes [9, 10, 11, 12, 13].

Accurate imaging is critical in diagnosing cervical cancer recurrence, guiding clinical decision‐making, and enhancing treatment plans. To diagnose recurring disease, a variety of imaging techniques have been utilized, including ultrasound, computed tomography (CT), and magnetic resonance imaging (MRI). However, these conventional methods have challenges while distinguishing posttreatment alterations from genuine malignancy, particularly in cases of modest recurrence or tiny metastatic deposits [14, 15, 16]. In contrast, 18F‐Fluorodeoxyglucose positron emission tomography‐computed tomography (18F‐FDG PET‐CT) provides both anatomical and functional information by detecting areas of increased glucose metabolism, which is found in malignant cells [17, 18, 19]. 18F‐Fluorodeoxyglucose (F‐18 FDG) is a radioactive tracer used in nuclear medicine to detect cancerous tumors that would have been overlooked or difficult to characterize using other imaging methods [17]. This hybrid imaging approach has demonstrated excellent sensitivity and specificity in detecting recurrent cervical cancer, particularly distant metastases. F‐18 FDG PET‐CT is an effective technique for following the incidence of suspected cervical cancer recurrence both locally and distantly because it provides whole‐body imaging in a single scan [19, 20, 21].

Recurrent cervical cancer usually follows three main metastatic pathways: local recurrence in the vaginal vault, cervix, or uterus; lymphatic dissemination to pelvic, para‐aortic, and supraclavicular lymph nodes; and distant hematogenous metastases to organs like the lungs, liver, and bones [10]. The histological subtypes of cervical cancer, primarily squamous cell carcinoma (SCC) and adenocarcinoma (ADC), have unique recurrence patterns. Squamous cell carcinoma is more prone to spread lymphatically, notably to the pelvic and para‐aortic lymph nodes, whereas adenocarcinoma spreads hematogenously to distant organs such as the lungs and liver [11, 22]. Understanding these metastatic pathways is critical for deciding on suitable follow‐up techniques and the most effective treatment options for recurrent disease. Although the increasing use of PET‐CT in oncology, there has been a lack of extensive research into its role in detecting both local and distant cervical cancer recurrence in resource‐constrained settings. Access to advanced imaging techniques is generally restricted in Pakistan, where cervical cancer remains a serious public health concern [23, 24]. Early detection of recurrent cervical cancer significantly improves the survival rate, increased treatment options and allows for prompt management of symptoms associated with recurrence, improving patient quality of life in patients with recurrent cervical cancer. It is used to know about the metastatic condition, tumor extent, lymph node extension, and tumor size. Imaging determines the staging, which in turn determines the best therapeutic method that is implemented to treat the cervical cancer [25, 26, 27].

This cross‐sectional study aimed to evaluate the frequency of local and distant metastases detected on ^18F‐FDG PET‐CT in patients with suspected cervical cancer recurrence. In addition, recurrence patterns were analyzed according to histological subtype and primary treatment modality. These insights may help refine follow‐up strategies for high‐risk patients, enabling earlier intervention and improved clinical outcomes.

## 2 Materials and Methods

### 2.1 Study Design, Setting, Duration, Sampling Approach and Sample Size

This was an observational retrospective cohort research conducted at the Institute of Nuclear Medicine and Oncology Lahore (INMOL) in Lahore, Pakistan. The study lasted 7 months, from July 4th to January 31st, 2025. A non‐probability purposive sampling technique was employed to select patients, ensuring that only relevant cases that met the inclusion criteria were included and that the study remained focused on cervical cancer recurrence.

The sample size was calculated using the single‐proportion formula; *n* = *Z* ^*2*^ *× P × (1* − *P)/d* ^*2*^ [28], where *Z* is the *z*‐score for a 95% confidence level (1.96), *P* is the expected prevalence of recurrence, and d is the desired margin of error. Using a conservative prevalence estimate of 50% to maximize variance [*P*(1 − *P*) = 0.25] and a 10% margin of error (*d* = 0.10), the required sample size was 97. To account for potential incomplete records or missing data, the sample size was increased by 20%, resulting in a target of approximately 117 patients. For operational feasibility, this was rounded to 120 patients, providing sufficient precision to estimate recurrence rates and compare histological subgroups (SCC/ADC).

### 2.2 Ethical Considerations

The study was conducted in accordance with the Declaration and Ethical Guidelines of Helsinki for studies involving humans [29] and approved by the Institutional Review Board of the Faculty of Allied Health Sciences, the University of Lahore, Lahore, Punjab, Pakistan (Reference No: REC‐UOL‐1763‐07‐2024) on July 1st, 2024. Informed consent was waived by the Institutional Review Board due to the retrospective nature of the study. During the preparation of this manuscript, the author used ChatGPT (OpenAI, version 4.5o) to assist with improving clarity, grammar, and overall readability. The author reviewed and edited the output and takes full responsibility for the content of this publication.

### 2.3 Study's Inclusion and Exclusion Criteria

This study included patients who had undergone 18F‐FDG PET‐CT at INMOL Hospital between July 2, 2024, and January 31, 2025. The patients who had previously undergone cervical cancer treatment via surgery, radiation, chemotherapy, or a combination of these treatment approaches were also included. Additionally, patients who obtained a diagnostic computed tomography scan within one to 2 months of the PET‐CT scan were included in this study. The study's exclusion criteria included patients who had incomplete or missing data, like clinical or histological information, which hindered the evaluation of the recurring patterns. Patients who were being treated for cervical cancer at the time of the imaging were also excluded because this study was focused only on posttreatment cervical cancer recurrence.

In this retrospective cohort, ^18F‐FDG PET‐CT was not performed routinely for all posttreatment patients. Instead, referral for PET/CT was determined by clinical suspicion of recurrence (e.g., new symptoms, abnormal clinical examination) or inconclusive findings on conventional imaging such as ultrasound, CT, or MRI. This approach reflects institutional practice at INMOL, where PET/CT is used selectively in high‐risk or diagnostically uncertain cases rather than as part of universal surveillance.

### 2.4 Imaging Protocol

All 18F‐FDG PET‐CT scans were carried out with a GE Discovery 64‐slice PET‐CT scanner, following standard nuclear medicine imaging techniques. Patients were told to fast for at least 4‐5 h before the scan to achieve maximum FDG uptake and minimal background activity. Before the FDG injection, blood glucose levels were tested to ensure that they were less than 180 mg/dL to avoid false‐positive results caused by abnormal glucose metabolism. Patients were given an intravenous injection of 18F‐FDG at a dose of 3–5 MBq/kg body weight. Afterwards, the radioactive tracer injection, patients relaxed in a low‐stimulation environment for at least 55 min to ensure adequate tracer dispersion. To minimize urinary background activity, patients were asked to drink at least 1 liter of water orally for hydration and urinate before the scan.

The PET‐CT scans were performed from head to mid‐thigh to capture the whole‐body imaging in a single session. All the patients were positioned supine with arms lifted over their heads. A low‐dose computerized axial tomography (CAT) scan (120 kVp, 100–200 mAs, 3.75 mm slice thickness) was first obtained for anatomical localization and attenuation correction. PET pictures were then captured in 3D mode for 3 min each bed position, covering the whole scan range in about 20 min. The highest standardized uptake value (SUVmax) of all suspicious lesions was computed for semi‐quantitative analysis. Patients were observed for 15–20 min after imaging to ensure no adverse reactions to the contrast medium occurred. There were no severe complications reported, and patients were discharged following regular post‐scan care.

### 2.5 Data Collection

The study was conducted after getting ethical approval from the institutional ethical committee. As this was an observational retrospective study, patient consent was not sought. Medical records, imaging reports, and histological findings of patients with probable cervical cancer recurrence were obtained from the hospital's nuclear medicine and radiology departments. The PET‐CT pictures were analyzed by two independent nuclear medicine physicians and radiologists who were unaware of the clinical results. FDG uptake (SUVmax values) and lesion distribution were examined. Patients' demographics, cervical cancer histological subtypes, past medical history, imaging results, and metastatic involvement were all gathered using a standardized data collection sheet.

### 2.6 Statistical Analysis

The data were analyzed using the Statistical Package for the Social Sciences (SPSS), version 26.0. Continuous variables were expressed as mean ± standard deviation (SD), and categorical variables as frequencies and percentages. The Chi‐square test was applied to compare recurrence rates between SCC and ADC and to assess associations between histological type and metastatic patterns. Odds ratios (OR) with 95% confidence intervals (CI) were calculated. A *p*‐value ≤ 0.05 was considered statistically significant. Results were presented in tables and supported by graphical illustrations prepared in Excel.

## 3 Results

This table summarizes the demographic and clinical features of the 120 cervical cancer patients who participated in the study. The patients' average age was 48.7 ± 10.2 years, with a range of 23–74 years. The majority of cases were SCC (73.3%), with ADC accounting for 26.7% of the patients. According to the International Federation of Gynecology and Obstetrics (FIGO) staging system, 48.3% of patients were diagnosed in early stages (I–II) and 51.7% in advanced stages (III–IV). Lymphovascular invasion (LVI) was detected in 32.5% of cases, indicating that a subset of patients had a significant risk of cervical cancer invasion. Among the study's participants, 30.8% of the patients were smokers, which is an established risk factor for cervical cancer progression, and 54.2% of the patients had gone through menopause, which could have an impact on tumor biology and treatment response. Regarding primary treatment, the majority received definitive chemoradiation (56.7%), followed by combined therapy (22.5%) and surgery alone (20.8%). This distribution reflects institutional practice, where chemoradiation is preferred for advanced‐stage cases, while surgery is typically reserved for early‐stage disease (Table 1).

**Table 1:** Descriptive statistics of cervical cancer patients (*N* = 120).

Variable	Category	*n* (%)
Gender	Female	120 (100%)
Age (years)	Mean ± SD	48.7 ± 10.2
	Minimum – Maximum	22−75
Histological type	Squamous Cell Carcinoma	88 (73.3%)
	Adenocarcinoma	32 (26.7%)
FIGO stage	Early‐stage (I–II)	58 (48.3%)
	Advanced‐stage (III–IV)	62 (51.7%)
Lymphovascular invasion (LVI)	Present	39 (32.5%)
	Absent	81 (67.5%)
Smoking history	Smoker	37 (30.8%)
	Nonsmoker	83 (69.2%)
Menopausal status	Premenopausal	55 (45.8%)
	Post‐Menopausal	65 (54.2%)
Primary treatment modality	Surgery alone (radical hysterectomy ± lymphadenectomy)	25 (20.8%)
	Definitive chemoradiation (CCRT ± brachytherapy)	68 (56.7%)
	Combined therapy (surgery + adjuvant chemoradiation)	27 (22.5%)

Table 2 demonstrates the total frequency distribution of recurrence in cervical cancer patients according to histological type SCC versus ADC. The total recurrence rate was 65.0% (*n* = 78), with 35.0% (*n* = 42) of patients remaining recurrence‐free following cervical cancer treatment. Among the study's participants, SCC patients showed a higher rate (43.3%) than ADC patients (21.7%). There were more cervical cancer recurrence‐free patients in SCC (30.0%) than in ADC (5.0%). The statistical analysis revealed a significant difference in recurrence rates between both histological categories. The odds ratio of 0.33 indicates that SCC patients had a 67% reduced chance of recurrence compared to ADC patients. The data show that ADC patients had a much higher risk of recurrence, underlining the importance of tighter follow‐up, intensive monitoring, and potentially more aggressive surveillance approaches in ADC cases for timely and precise diagnosis of cervical cancer reoccurrence.

**Table 2:** Frequency distribution of total recurrence of cervical cancer on FDG PET‐CT.

Recurrence	Squamous Cell Carcinoma (SCC)		Adenocarcinoma (ADC)		Total	Statistical Analysis
	Frequency (*n*)	Percentage (%)	Frequency (*n*)	Percentage (%)		
Present	52	43.3%	26	21.7%	78 (65.0%)	χ² = 4.14 *p* = 0.041 OR = 0.33 (95% CI: 0.12–0.89)
Absent	36	30.0%	6	5.0%	42 (35.0%)	
Total	88	73.3%	32	26.7%	120 (100%)	

As shown in the Tables 3, 33.3% (*n* = 40) of the total patients experienced local recurrence, with 19.2% of SCC cases and 14.2% of ADC cases indicating recurrence. A chi‐squared test found a statistically significant correlation between histological type and local recurrence. The OR suggests that SCC patients had a 69% lower risk of local recurrence than ADC patients. This study shows that ADC patients may have a higher risk of local recurrence, which could affect posttreatment surveillance and management strategies.

**Table 3:** Frequency distribution of local recurrence of cervical cancer on FDG PET‐CT.

Local Recurrence	Squamous Cell Carcinoma (SCC)		Adenocarcinoma (ADC)		Total	Statistical Analysis
	Frequency (*n*)	Percentage (%)	Frequency (*n*)	Percentage (%)		
Present	23	19.2%	17	14.2%	40 (33.3%)	χ² = 6.53 *p* = 0.011 OR = 0.31 (95% CI: 0.13–0.72)
Absent	65	54.2%	15	12.5%	80 (66.7%)	
Total	88	73.3%	32	26.7%	120 (100%)	

Table 4 depicts the frequency distribution of lymph node (LN) metastasis on FDG PET‐CT among cervical cancer patients with recurrence. It demonstrates that the neck lymph node metastasis was found in 10.8% (*n* = 13) of patients, with 8.3% SCC and 2.5% ADC patients. Chi‐square analysis revealed no statistically significant difference between SCC and ADC. The odds ratio reveals a slightly increased risk in SCC patients, but the confidence interval exceeds 1.0, indicating a lack of statistical significance. These findings suggest that SCC and ADC patients have a comparable risk of acquiring neck lymph node metastases.

**Table 4:** Frequency distribution of lymph nodes (LN) metastasis on FDG PET‐CT.

Lymph Node Metastases		Squamous Cell Carcinoma (SCC)		Adenocarcinoma (ADC)		Total	Statistical Analysis
		Frequency (*n*)	Percentage (%)	Frequency (*n*)	Percentage (%)		
Neck LN Metastasis	Present	10	8.3%	3	2.5%	13 (10.8%)	χ² = 0.00 *p* = 1.00 OR = 1.24 (95% CI: 0.32–4.82)
	Absent	78	65.0%	29	24.2%	107 (89.2%)	
Total		88	73.3%	32	26.7%	120 (100%)	
Chest LN Metastasis	Present	42	35.0%	7	5.8%	49 (40.8%)	χ² = 5.47 *p* = 0.019 OR = 3.26 (95% CI: 1.28–8.32)
	Absent	66	38.3%	25	20.8%	71 (59.2%)	
Total		88	73.3%	32	26.7%	120 (100%)	
Abdominal LN Metastasis	Present	21	17.5%	9	7.5%	30 (25.0%)	χ² = 0.06 *p* = 0.81 OR = 0.80 (95% CI: 0.32–2.00)
	Absent	67	55.8%	23	19.2%	90 (75.0%)	
Total		88	73.3%	32	26.7%	120 (100%)	
Pelvic LN Metastasis	Present	14	11.7%	12	10.0%	26 (21.7%)	χ² = 5.24 *p* = 0.022 OR = 0.32 (95% CI: 0.13–0.79)
	Absent	74	61.7%	20	16.7%	94 (78.3%)	
Total		88	73.3%	32	26.7	120 (100%)	

As Table 4 shows, the chest lymph node metastasis was observed in 40.8% (*n* = 49) of cases, making it the most common lymphatic spread site. Compared to 5.8% of ADC patients, 35.0% of SCC patients experienced metastases. Histological type and chest lymph node metastasis were significantly correlated, according to chi‐squared analysis. The odds ratio indicates that SCC patients were 3.26 times more likely to develop chest lymph node metastasis compared to ADC patients. These outcomes emphasize the importance of close monitoring of SCC patients for chest lymph node involvement.

This study indicates that abdominal lymph node metastasis was observed in 25.0% (*n* = 30) of patients, with 17.5% of SCC cases and 7.5% of ADC cases. However, the difference was not statistically significant. The odds ratio indicates a slightly reduced risk in SCC patients; nevertheless, the confidence interval includes 1.0, making the difference clinically insignificant. These results suggest that abdominal lymph node metastasis occurs with similar frequency in both SCC and ADC patients [Table 4].

Furthermore, Table 4 demonstrates that 21.7% (*n* = 26) of patients had pelvic lymph node metastases, with 11.7% SCC and 10.0% ADC patients. The chi‐square test revealed a statistically significant correlation. The odds ratio indicates that SCC patients were considerably less likely to have pelvic lymph node metastases than ADC individuals. These outcomes suggest that ADC may metastasize more easily to the pelvic lymph nodes, which may alter treatment approaches.

Table 5 demonstrates the frequency distribution of visceral metastasis on FDG PET‐CT. Lung metastasis was found in 17.5% (*n* = 21) of patients, including 11.7% SCC and 5.8% ADC instances. However, Chi‐square analysis revealed no significant differences between SCC and ADC. The odds ratio of 0.68 implies a slightly decreased risk in SCC patients, but the confidence interval exceeds 1.0, indicating a lack of statistical significance. These outcomes indicate that lung metastases occur at equal rates in both histological types, and it should be monitored in the women with cervical cancer.

**Table 5:** Frequency distribution of visceral metastases of cervical cancer on FDG PET‐CT.

Visceral metastases		Squamous Cell Carcinoma (SCC)		Adenocarcinoma (ADC)		Total	Statistical Analysis
		Frequency (*n*)	Percentage (%)	Frequency (*n*)	Percentage (%)		
Lung metastasis	Present	14	11.7%	7	5.8%	21 (17.5%)	χ² = 0.24 *p* = 0.62 OR = 0.68 (95% CI: 0.25–1.86)
	Absent	74	61.7%	25	20.8%	99 (82.5%)	
Total		88	73.3%	32	26.7%	120 (100%)	
Liver metastasis	Present	12	10.0%	5	4.2%	17 (14.2%)	χ² = 0.00 *p* = 1.00 OR = 0.85 (95% CI: 0.27–2.64)
	Absent	76	63.3%	27	22.5%	103 (85.8%)	
Total		88	73.3%	32	26.7%	120 (100%)	

In addition, Table 5 indicates that liver metastasis was found in 14.2% (*n* = 17) of patients, comprising 10.0% SCC and 4.2% ADC instances. However, a Chi‐Square study revealed no statistically significant correlation. The odds ratio implies there is no significant difference between SCC and ADC patients. These findings suggest that liver metastasis is equally common in both histological categories (SCV and ADC).

Table 6 shows the frequency distribution of skeletal metastasis on FDG PET‐CT. Axial skeletal metastasis was observed in 8.3% (*n* = 10) of patients, but only in SCC instances. No ADC patients developed bone metastases, resulting in an unclear odds ratio. The chi‐square test was not statistically significant. In this study, ADC had no cases involving axial skeleton metastases; hence, the odds ratio is ∞ (95% CI: NaN ‐ ∞), indicating that the association cannot be statistically quantified. However, the tendency indicates that SCC may have a more risk of axial skeletal metastasis than ADC, while additional research is needed to validate this finding.

**Table 6:** Frequency Distribution of Axial Skeletal Metastasis on FDG PET‐CT.

Skeletal Metastasis		Squamous Cell Carcinoma (SCC)		Adenocarcinoma (ADC)		Total	Statistical Analysis
		Frequency (*n*)	Percentage (%)	Frequency (*n*)	Percentage (%)		
Axial Skeletal Metastasis	Present	10	8.3%	0	0.0%	10 (8.3%)	χ² = 2.62 *p* = 0.106 OR = ∞ (95% CI: NaN – ∞) (since ADC had 0 cases, OR is undefined)
	Absent	78	65.0%	32	26.7%	110 (91.7%	
Total		88	73.3%	32	26.7	120 (100%)	

Table 7 shows recurrence patterns according to the type of initial therapy. Patients treated with surgery alone (radical hysterectomy ± lymphadenectomy) demonstrated the lowest recurrence rate (44.0%), whereas those managed with definitive chemoradiation (70.6%) or combined therapy (70.4%) showed considerably higher recurrence proportions. The overall association between treatment modality and recurrence was statistically significant (χ² = 6.12, df = 2, *p* = 0.047).

**Table 7:** Recurrence of cervical cancer by primary treatment modality on ^18F‐FDG PET‐CT (*N* = 120).

Primary treatment modality	Typical Clinical Indication (Stage/Case Mix)	Recurrence Present *n* (%)	Recurrence Absent *n* (%)	Total Patients *n* (%)
Surgery alone (radical hysterectomy ± lymphadenectomy)	Early‐stage (FIGO IA–IIA), low‐risk features	11 (44.0%)	14 (56.0%)	25 (20.8%)
Definitive chemoradiation (CCRT ± brachytherapy)	Locally advanced disease (FIGO IIB–IVA)	48 (70.6%)	20 (29.4%)	68 (56.7%)
Combined therapy (surgery + adjuvant chemoradiation)	Early/intermediate stages with high‐risk pathology (e.g., LVSI, positive margins, nodal involvement)	19 (70.4%)	8 (29.6%)	27 (22.5%)
Total	—	78 (65.0%)	42 (35.0%)	120 (100%)

**Figure 1:**
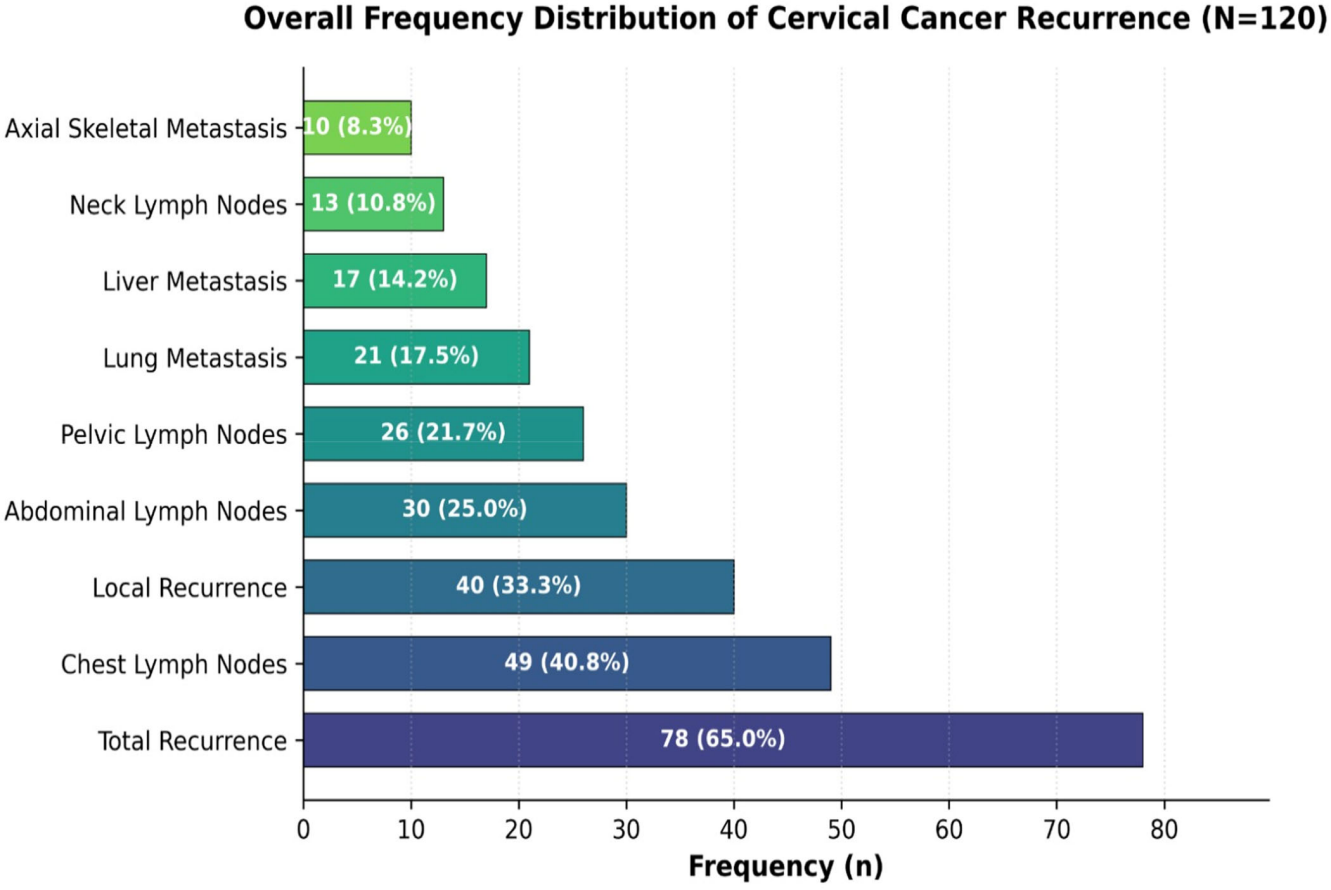
Overall distribution of cervical cancer recurrence among the study's participants.

Figure 1 depicts the overall frequency distribution of cervical cancer recurrence and metastasis diagnosed using FDG PET‐CT. Total recurrence: 65.0% (*n* = 78) of all participants experienced recurrence, indicating a high disease burden in this population. Local recurrence occurred in 33.3% (*n* = 40) of patients, highlighting the significance of regular follow‐ups in posttreatment patients. The chest was the most common site for lymph node involvement (40.8%). Lymph node metastases occurred in the abdomen, pelvis, and neck with rates of 25.0%, 21.7%, and 10.8%, respectively. Visceral and skeletal metastases: 17.5% of patients had lung metastases, whereas 14.2% had liver metastases. Axial skeletal metastases were the least prevalent (8.3%), indicating that distal bone spread is extremely uncommon. These findings highlight the significance of FDG PET‐CT for diagnosing cervical cancer recurrence and distant metastases, which may assist in guiding treatment planning and surveillance strategies.

## 4 Discussion

Cervical cancer recurrence remains a serious clinical problem needing early and accurate identification, which is essential for guiding timely therapeutic interventions. F‐18 FDG PET‐CT has proven to be a highly effective imaging modality, capable of detecting recurrence even when other imaging techniques, such as CT or MRI, yield inconclusive results. In this study, recurrence rates, metastatic patterns, histological differences, and treatment‐related outcomes were evaluated in posttreatment cervical cancer patients.

### 4.1 Interpretation of Findings

This cross‐sectional analysis of 120 treated cervical cancer patients revealed a high overall recurrence rate of 65.0%. This reflects a substantial disease burden in the study population, which likely included many advanced‐stage cases, as suggested by prior reports indicating recurrence may reach 28%–70% in stage IIB–IV cancers [30]. The majority of cases in the current study were SSC (73.3%), with ADC comprising 26.7%. In absolute terms, more recurrences were observed in SCC (43.3% of patients) than ADC (21.7%). However, the OR analysis indicated that ADC patients had a significantly higher risk of recurrence (OR ≈ 3.03) than SCC patients. This aligns with known histologic behavior: adenocarcinomas tend to be more aggressive with greater propensity for distant spread. Thus, ADC patients in this cohort may require particularly vigilant follow‐up and intensive monitoring.

Local (pelvic) recurrences were identified on PET/CT in 33.3% of patients (19.2% of SCC and 14.2% of ADC cases). The high rate of locoregional relapse underscores the need for careful posttreatment surveillance of the cervix and vaginal vault. Overall, lymphatic metastases were common, with chest nodes (likely representing mediastinal or supraclavicular involvement) being most frequent (40.8% of patients), followed by abdominal nodes (25.0%) and pelvic nodes (21.7%). Notably, SCC patients were much more likely than ADC patients to have chest nodal metastases (35.0% vs. 5.8%, OR ≈ 3.26), whereas ADC showed a tendency for more pelvic nodal spread (10.0% vs. 11.7%). These findings are consistent with the canonical metastatic pathways: SCC typically disseminates via lymphatics (often to pelvic/paraaortic and even supraclavicular nodes), while ADC more often spreads hematogenously. Visceral metastases also followed expected patterns: lung (17.5% of patients) and liver (14.2%) were the most common organ sites. Bone involvement was relatively rare (8.3%), seen only in SCC cases, suggesting that axial skeletal metastasis was an uncommon event in this cohort. The current study's findings highlight that recurrence was significantly lower in surgically treated patients compared with those receiving chemoradiation or combined therapy, highlighting the influence of initial treatment modality on long‐term outcomes.

These PET/CT findings have important clinical relevance. They demonstrate that treated cervical cancer patients frequently harbor occult recurrent disease both locally and distantly. The detection of local recurrence in one‐third of patients emphasizes that surveillance should include sensitive imaging modalities. Likewise, the high rates of nodal and visceral metastases indicate that restaging must be whole‐body in scope. Early identification of these recurrences can allow timely initiation of salvage treatments or systemic therapy. In summary, the recurrence patterns in this study highlight the multifocal nature of relapse and confirm that ^18F‐FDG PET/CT is capable of comprehensively mapping both locoregional and distant disease.

### 4.2 Comparison With Existing Literature

The observed recurrence rate (65%) is higher than typical figures cited in the literature, but this likely reflects the high‐risk population included in this study (patients referred for PET/CT on suspicion of recurrence). In general populations, recurrence after standard therapy is often reported between 10% and 50% [31], varying by stage. For example, early‐stage (FIGO IB–IIA) disease recurs in about 11%–22% of cases, while more advanced stages (IIB–IVA) recur in 28%–64%, and stage III–IVB may approach 70% [30]). The high recurrence rate in this cohort is thus consistent with an advanced‐case mix. The particularly aggressive nature of ADC is well‐documented. Jung et al. found that usual‐type cervical adenocarcinoma had a significantly worse prognosis and a higher risk of distant recurrence than SCC [32], which corresponds with the finding of this study that ADC carried an elevated recurrence risk despite its lower absolute frequency. Furthermore, Miccò et al. noted that recurrent cervical cancer portends poor survival [10] —a conclusion supported by the emphasis on aggressive surveillance in this analysis.

Regarding metastatic patterns, the results of this study are broadly in line with other reports of cervical cancer spread. A recent Chinese analysis of 572 metastatic cervical cancer patients showed lung as the most common site (41.3%), followed by bone (15.2%) and liver (11.5%) [33]. Although the cohort was smaller and not limited to confirmed metastatic cases, this study similarly found the lung (17.5%) and liver (14.2%) to be frequent visceral sites, while the bone metastasis rate was lower. The predominance of lymphatic spread in chest (mediastinal/supraclavicular) and abdominal nodes mirrors known pathways of advanced cervix cancer. In a Pakistani series of 52 cervical carcinoma cases, the vast majority (92.8%) were SCC, underscoring that SCC is the dominant histology in this region. In that study, most presented at FIGO II–III, aligning with the notion of late‐stage presentation in Pakistan [34].

Stratification by treatment modality demonstrated that recurrence was least frequent among patients managed surgically (44.0%), while both chemoradiation (70.6%) and combined therapy (70.4%) groups exhibited markedly higher recurrence rates, with an overall significant association. This finding is consistent with prior evidence: patients with early‐stage disease who undergo radical hysterectomy generally have the lowest recurrence risk (typically < 25%) [35, 36]. In contrast, those treated with definitive chemoradiation for locally advanced disease (FIGO IIB–IVA) experience recurrence in 25%–64% of cases [37, 38]. Patients requiring adjuvant chemoradiation after surgery typically harbor adverse pathological features (e.g., positive nodes, lymphovascular invasion, or involved margins), explaining their higher recurrence burden [39]. These results therefore align with published literature and underscore that disease stage and baseline pathology, rather than treatment intensity alone, drive recurrence risk. These findings support the role of PET/CT surveillance particularly in high‐risk patients treated with radiation‐based modalities.

In summary, the findings of this study are consistent with both global and regional evidence. SSC remains the predominant subtype, while ADC carries a higher risk of recurrence. The most frequent metastatic sites were the lungs, liver, and regional lymph nodes. Recurrence was also significantly associated with primary treatment modality, being lowest after surgery and highest among patients treated with radiation‐based or combined therapies.

### 4.3 Clinical and Public Health Implications

These findings highlight the value of ^18F‐FDG PET/CT in the management of cervical cancer survivors. PET/CT has well‐documented high diagnostic performance for detecting recurrence. A meta‐analysis reported pooled sensitivity of 87% for distant and 82% for locoregional recurrence metastasis [40]. Likewise, Mittra et al. demonstrated “favorable efficacy of ^18F‐FDG PET/CT for identification of residual/recurrent cervical cancer, as well as for localization of distant metastases” [19]. In practice, PET/CT can reveal otherwise occult lesions – for example, in this series it uncovered extensive chest nodal and visceral disease in many patients – enabling clinicians to tailor treatment plans accordingly. In the Mittra study, PET/CT findings prompted changes in management for every patient examined [19]. Such changes might include shifting from presumed local therapy to systemic chemotherapy, adding radiotherapy fields, or pursuing surgical salvage. By defining the full extent of disease, PET/CT guides selection of appropriate salvage strategies (e.g. pelvic exenteration for isolated local recurrence *vs.* chemotherapy for distant metastases) and spares patients from futile aggressive interventions.

In a broader sense, earlier and more accurate detection of recurrence can improve patient outcomes. Detecting relapse when disease burden is lower may allow more effective salvage (surgical resection of isolated metastasis or targeted radiation). It also permits timely initiation of palliative care measures to maintain quality of life. From a prognostic viewpoint, PET‐positive recurrences are known to predict worse survival [9, 19], so their identification can inform clinical counseling. In low‐resource settings such as Pakistan, the adoption of PET/CT must be balanced against cost and availability. Advanced imaging is limited in many centers, and routine follow‐up often relies on clinical exam or ultrasound. Nevertheless, this study suggests that the selective use of PET/CT – for example, in patients with high‐risk histology or equivocal conventional imaging – could yield high diagnostic value. It should be noted that at least one cost‐effectiveness analysis (from a high‐income context) found routine PET/CT surveillance to be not cost‐effective, with an extremely high cost per QALY gained [41]. However, that analysis excluded symptomatic or high‐risk patients; in practice, PET/CT is usually reserved for clinical suspicion of recurrence. More research is needed to determine the optimal use of PET/CT in resource‐limited health systems. In the meantime, health policy should consider strategies such as centralizing PET/CT services and prioritizing high‐yield cases.

Based on the findings of this study and prior literature, incorporation of whole‐body PET/CT into follow‐up protocols may be particularly useful for patients with high‐risk clinical or pathological features. Potential referral criteria include: advanced FIGO stage at diagnosis (III–IV), adenocarcinoma histology, presence of lymphovascular space invasion, positive or close surgical margins, persistent or rising serum tumor markers, or equivocal findings on conventional imaging. These parameters can help prioritize PET/CT use in resource‐limited settings, ensuring that the modality is applied to patients most likely to benefit from early detection of recurrence.

From a public health standpoint, these results reinforce the urgency of preventive measures (screening, HPV vaccination) to reduce advanced disease and recurrence. Meanwhile, optimizing follow‐up protocols for treated patients (potentially incorporating PET/CT where available) may allow earlier detection of relapse, which could translate into better survival in this otherwise high‐risk population.

### 4.4 Limitations and Future Directions

This study has several limitations. As a single‐center, retrospective analysis with a modest sample size (*n* = 120), this study's findings may not be fully generalizable. The purposive sampling (patients referred for PET/CT) introduces selection bias toward higher‐risk or symptomatic individuals, which likely inflated the observed recurrence rate compared to an unselected cohort. Histological confirmation of recurrence was not available for all lesions; PET/CT findings could include false positives (inflammation or infection). The cross‐sectional design also precludes assessment of temporal outcomes, as patients were not followed longitudinally to measure survival or time to progression. Additionally, because all imaging and interpretation were performed at a single institution, interobserver variability was not assessed. These constraints should temper over‐interpretation of the current study's results.

Future research should focus on conducting large, multicenter prospective studies to validate these recurrence rates and metastatic patterns across diverse populations. Longitudinal follow‐up is needed to assess the prognostic impact of early recurrence detection on survival and quality of life. Expanding research to include different geographic regions, particularly within South Asia, will help identify potential variations in recurrence behavior. Additionally, integrating novel imaging techniques such as PET/MRI, advanced radiotracers, and combining PET‐CT findings with serum biomarkers could enhance the sensitivity and specificity of recurrence detection, ultimately guiding more individualized surveillance and treatment strategies.

## 5 Conclusion

This study demonstrates that ^18F‐FDG PET‐CT is a valuable imaging modality for detecting recurrence and metastases in posttreatment cervical cancer patients. The overall recurrence rate was high, with adenocarcinoma showing a greater relative risk compared to squamous cell carcinoma. PET‐CT effectively identified both local and distant disease, with chest lymph nodes, abdominal nodes, lungs, and liver being the most frequent metastatic sites, while axial skeletal involvement was rare. Importantly, recurrence was significantly associated with treatment modality: patients treated with surgery alone experienced the lowest recurrence, whereas those managed with chemoradiation or combined therapy had substantially higher rates. These findings align with international literature and underscore that disease stage and pathological risk factors, rather than treatment intensity alone, largely determine recurrence risk. These findings highlight the importance of incorporating whole‐body PET‐CT into follow‐up protocols for high‐risk patients, enabling early detection and tailored management strategies to improve clinical outcomes.

## Author Contributions

**Babar Ali:** conceptualization, investigation, writing – original draft, writing – review and editing, visualization, methodology, validation, software, formal analysis, project administration, data curation, supervision, resources.

## Consent

The author has read and approved the final version of the manuscript. The corresponding author, Babar Ali, had full access to all of the data in this study and takes complete responsibility for the integrity of the data and the accuracy of the data analysis.

## Conflicts of Interest

The authors declare no conflicts of interest.

## Declarations

The authors have nothing to declare.

## Transparency Statement

The lead author Babar Ali affirms that this manuscript is an honest, accurate, and transparent account of the study being reported; that no important aspects of the study have been omitted; and that any discrepancies from the study as planned (and, if relevant, registered) have been explained.

## Data Availability

The data that support the findings of this study are available on request from the corresponding author. The data are not publicly available due to privacy or ethical restrictions.
